# Cine phase-contrast magnetic resonance imaging evaluation of cerebrospinal fluid flow dynamics in healthy pediatric subjects

**DOI:** 10.1590/0100-3984.2021.0120

**Published:** 2022

**Authors:** Karen Sousa Plata, Gerardo Cruz, Hector Lezcano

**Affiliations:** 1 Hospital Santo Tomás, Panama City, Panama.; 2 Dr. Arnulfo Arias Madrid Hospital, Panama City, Panama.

**Keywords:** Cerebrospinal fluid, Magnetic resonance imaging, cine, Cerebral aqueduct, Hydrodynamics, Líquido cerebrospinal, Imagem cinética por ressonância magnética, Aqueduto do mesencéfalo, Hidrodinâmica

## Abstract

**Objective:**

To evaluate cerebrospinal fluid dynamics, using cine phase-contrast magnetic resonance imaging (cine-PC MRI), in healthy pediatric subjects, determining the normal flow values in this population, as well as identifying differences related to age, sex, and body surface area.

**Materials and Methods:**

This was a descriptive cross-sectional study involving 32 healthy children and adolescents, in whom the flow of cerebrospinal fluid through the cerebral aqueduct was evaluated quantitatively with cine-PC MRI. We used specialized software to analyze the images obtained with cine-PC MRI, drawing a region of interest on the aqueduct. A flow-time curve was obtained, as were automated measurements of the various parameters.

**Results:**

The following normal (mean) values were obtained: net flow, 1.10 ± 0.99 mL/m; stroke volume, 12.2 ± 10.1 µL/cycle; mean velocity, 0.72 ± 1.00 cm/s; peak systolic velocity, 5.28 ± 2.30 cm/s; peak diastolic velocity, 4.51 ± 1.77 cm/s. These values were not affected by age or sex. In addition, body surface area was not found to correlate significantly with mean velocity or stroke volume.

**Conclusion:**

In children and adolescents, the basic cerebrospinal fluid flow parameters, as determined by cine-PC MRI, appear to be independent of age and sex.

## Abstract (pt)

**Objetivo:**

Avaliar a dinâmica do fluxo do líquido cerebrospinal por cine-ressonância magnética com contraste de fase em crianças saudáveis, para determinar os valores normais de fluxo nesta população, bem como a diferença entre medições distintas de acordo com idade, sexo ou área da superfície corporal.

**Materiais e Métodos:**

Convocamos 32 crianças saudáveis em uma análise descritiva de prevalência para a avaliação quantitativa do fluxo do líquido cerebrospinal pelo aqueduto de Sylvius. A análise foi realizada por meio de *software* especializado, desenhando uma região de interesse no aqueduto. Uma curva de fluxo-tempo e medições automáticas de diversos parâmetros foram obtidas.

**Resultados:**

Os seguintes valores normais foram obtidos: fluxo efetivo de 1,10 ± 0,99 mL/m; volume médio de 12,2 ± 10,1 µL/ciclo; velocidade média de 0,72 ± 1,00 cm/s; pico de velocidade sistólica de 5,28 ± 2,30 cm/s; pico de velocidade diastólica de 4,51 ± 1,77 cm/s. Esses valores não foram afetados pela idade ou sexo das crianças. Além disso, não foi encontrada correlação significativa entre a área corporal, a velocidade média e o volume sistólico.

**Conclusão:**

De modo geral, os parâmetros básicos do fluxo do líquido cerebrospinal independem de idade e sexo em crianças.

## INTRODUCTION

Cerebrospinal fluid (CSF) is a transparent liquid, with a density of 1,003-1,008g/cm^3^ and a protein content lower than that of plasma; its main function is to provide protection to the brain and spinal cord through buoyancy^(1,2)^. The CSF also plays an important role in the homeostasis of the interstitial fluid of the brain parenchyma, as well as in regulation of neuronal function^(3)^. It is mainly produced by the epithelium of the choroid plexuses, located in the lumen of the lateral ventricles and in the fourth ventricle. After circulating through the ventricular system, subarachnoid spaces, and ependymal canal, CSF is absorbed into the venous system by the arachnoid granulations^(4,5)^. Additional sites of absorption include the spinal arachnoid villi near the epidural spinal veins and the meningeal sheaths of the spinal and cranial nerves. Lymphatics located proximal to arteries and nerves are also part of the CSF absorption mechanism^(6)^.

Evaluation of CSF flow dynamics, in adults and children, is an essential clinical tool for the assessment of various neurological diseases. Recently, cine phase-contrast magnetic resonance imaging (cine-PC MRI) has emerged as a useful technique for the measurement of multiple physiological parameters without the need for invasive maneuvers^(7,8)^. In addition, these measurements are useful in the preoperative evaluation of patients with idiopathic intracranial hypertension and in the postoperative follow-up after endoscopic third ventriculostomy^(9,10)^. Nevertheless, the applicability of flow measurements for the preoperative assessment of patients with normal-pressure hydrocephalus is controversial. Bradley et al.^(11)^ found that patients with a CSF stroke volume greater than 42 µL had a favorable response after shunting. However, in a more recent study, Kahlon et al.^(12)^ established that there is no clear relationship between stroke volume and clinical improvement after surgery.

Cine-PC MRI techniques have also demonstrated applicability in patients with arachnoid cysts, aiding in the differentiation of those that communicate with the ventricular system from those that do not^(13)^. In children, CSF flow studies are also clinically valuable, because of the high number of disorders affecting CSF kinetics, such as a Chiari I malformation and a brain tumor^(8,14)^. In addition, cine-PC MRI techniques facilitate the differentiation between communicating and non-communicating hydrocephalus in the pediatric population, as well as the distinction between hydrocephalus and *ex vacuo* ventricular dilatation, which is a challenging issue in pediatric neuroradiology^(15,16)^.

In order to perform appropriate evaluations of CSF flow dynamics in multiple pathological conditions, it is first essential to characterize the measurements in normal individuals. Unfortunately, most CSF characterization studies have been conducted in adults. The corresponding data for the pediatric population is scarce, worldwide and in Latin American countries. Therefore, we assessed CSF flow parameters using cine-PC MRI in healthy pediatric subjects, with the objective of characterizing the potential effects that age, sex, and body surface area (BSA) have on those parameters.

## MATERIALS AND METHODS

This was a descriptive cross-sectional study conducted over a three-month period in the radiology department of a tertiary care pediatric hospital. Pediatric subjects were recruited from among presumably healthy individuals undergoing cine-PC MRI of the brain during that period. The minimum sample size, calculated on the basis of the formula devised by Viechtbauer et al.^(17)^, using a confidence level of 80% and a probability of 0.05%, was 32 participants. The study was approved by the bioethics committee of the hospital. The parents or legal guardians of the subjects gave written informed consent.

All of the subjects were between 1 month and 14 years of age. Subjects with a CSF pathology, a structural brain/spinal pathology (known or suspected), or a history of head trauma were excluded, as were those in whom intra- or extra-axial lesions, ventriculomegaly, or alterations in the craniocervical junction were observed during the study. The cases were stratified by age group: 1-12 months (infants); 1-5 years (toddlers and preschool children); 5-11 years (children); and 12-14 years (adolescents).

All images were obtained in a 1.5-T MRI scanner (Optima MR450w; GE Healthcare, Milwaukee, WI, USA). The routine brain MRI procedure was performed, which involved acquiring the sequences required according to the indication of each study, using a standard head coil. Subsequently, a T2-weighted fluid-attenuated inversion recovery pulse sequence (Cube; GE Healthcare) was acquired in the axial plane for the evaluation of CSF characteristics (if it was not previously included), as were cine-PC MRI sequences with prospective cardiac triggering, a pulse oximeter being used for the qualitative and quantitative evaluation of CSF flow through the cerebral aqueduct. Velocity encoding was set at 20 cm/s. Table 1 summarizes the technical parameters used in order to acquire these MRI sequences.

**Table 1:** Technical parameters used in acquiring the MRI sequences.

Parameter	Cube T2 FLAIR	Cine-PC MRI	
		Quantitative	Qualitative
Plane	Sagittal	Axial	Sagittal
Repetition time (ms)	6,900	12.2	11.4
Echo time (ms)	99.9	7.4	30.0
Inversion time (ms)	1,840	-	-
Slice thickness (mm)	1.2	4.0	6.0
Matrix	224 × 224	256 × 256	256 × 256
Flip angle (°)	90	20	10
Field of view (mm)	240	180	240
Excitations (n)	1.0	4.0	1.0

FLAIR, fluid-attenuated inversion recovery.

**Figure 1:**
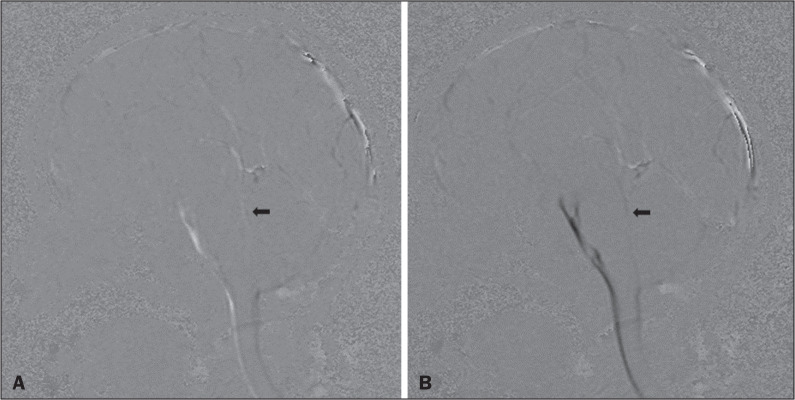
Qualitative sagittal cine-PC MRI sequences acquired during systole (A) and diastole (B) at the level of the cerebral aqueduct (arrows).

**Figure 2:**
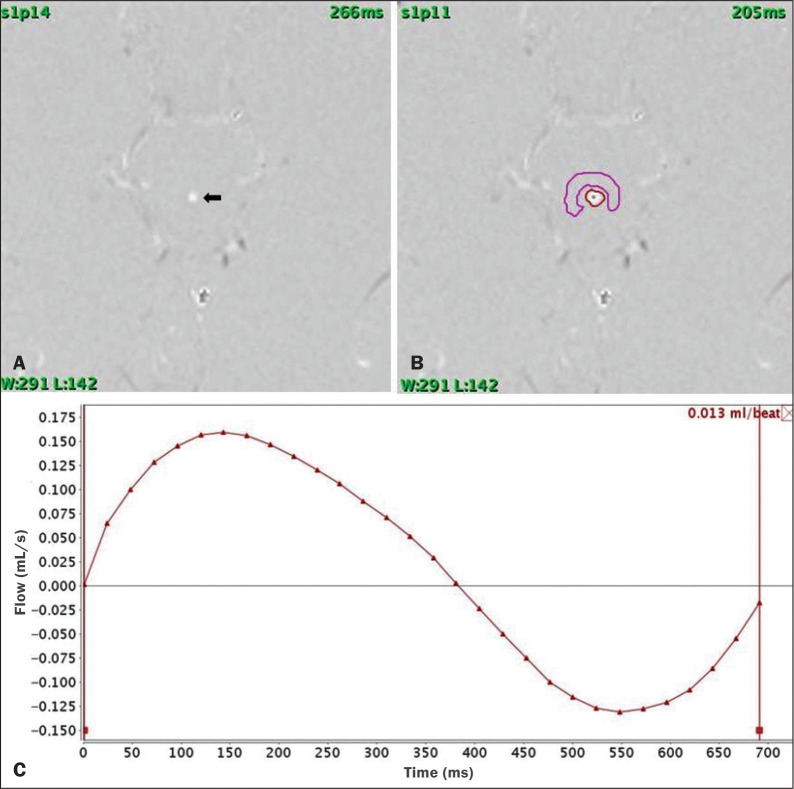
A: Magnified quantitative axial cine-PC MRI sequence at the level of the cerebral aqueduct (arrow). B: Region of interest over the cerebral aqueduct (red). Background subtraction area (fuchsia). C: Flow-time curve obtained during a cardiac cycle.

The images obtained were processed with the software READY View (GE Healthcare). The region of interest was drawn at the level of the cerebral aqueduct, as was an area of background subtraction in a magnified image, in the axial slices of the quantitative cine-PC MRI sequence (Figures 1 and 2). A flow-time curve was obtained during a cardiac cycle, as were multiple automated measurements, including net flow, stroke volume, mean velocity, peak systolic velocity, and peak diastolic velocity.

Statistical analysis was performed with the IBM SPSS Statistics software package, version 22.0 (IBM Corp., Armonk, NY, USA). Continuous quantitative variables are presented as means and standard deviations. Comparisons among and between age groups and sexes were made by using analysis of variance and Student’s t-tests. The correlation between BSA and stroke volume was evaluated with the Spearman correlation coefficient, whereas the Pearson coefficient was used in order to evaluate the correlation between BSA and mean velocity. Values of *p* < 0.05 were considered statistically significant.

## RESULTS

During the three-month study period, a total of 42 subjects underwent brain MRI including cine-PC MRI images for CSF flow evaluation. However, in seven subjects, there were artifacts that made it difficult to evaluate the images; in two, there were errors during the image processing; and one subject was found to have a brain tumor. Those subjects were excluded from the study, resulting in a final sample size of 32 subjects.

Of the 32 subjects included, 20 (62.5%) were female and 12 (37.5%) were male. Eleven (34.0%) of the subjects were in the 5- to 11-year age group, whereas the other three age groups accounted for seven subjects (22%) each. In the sample as a whole, the mean height was 116.0 ± 31.4 cm, the mean weight was 27.3 ± 16.2 kg, and the mean BSA was 0.90 ± 0.38 m^2^.

The data obtained through cine-PC MRI revealed the following mean values: net CSF flow, 1.10 ± 0.99 mL/m; stroke volume, 12.2 ± 10.1 µL/cycle; mean velocity, 0.72 ± 1.00 cm/s; systolic peak velocity, 5.28 ± 2.30 cm/s; and diastolic peak velocity, 4.51 ± 1.77 cm/s. Those values are further dissected by age group and sex in Tables 2 and 3, respectively.

**Table 2:** Normal values for CSF flow parameters in pediatric subjects, by age group.

Age group	n	Net flow (mL/m)	Stroke volume (µL)	Mean velocity (cm/s)	Peak systolic velocity (cm/s)	Peak diastolic velocity (cm/s)
1-12 months	7	1.24 ± 1.17	11.0 ± 10.6	1.21 ± 1.44	5.23 ± 2.16	-4.31 ± 1.44
1-5 years	7	1.44 ± 0.74	16.0 ± 8.9	0.67 ± 1.08	6.54 ± 1.93	-5.84 ± 1.34
5-11 years	11	0.89 ± 1.18	9.7 ± 10.3	0.42 ± 0.82	4.29 ± 2.62	-3.84 ± 2.10
12-14 years	7	0.96 ± 0.74	13.7 ± 11.2	0.70 ± 0.70	5.60 ± 1.96	-4.44 ± 1.42
Total	32	1.10 ± 0.99	12.2 ± 10.1	0.72 ± 1.00	5.28 ± 2.30	-4.51 ± 1.77

Results are expressed as mean ± standard deviation.

**Table 3:** Normal values for CSF flow parameters in pediatric subjects, by sex.

Sex	n	Net flow (mL/m)	Stroke volume (µL)	Mean velocity (cm/s)	Peak systolic velocity (cm/s)	Peak diastolic velocity (cm/s)
Male	12	1.32 ± 1.25	15.1 ± 12.2	0.94 ± 1.20	5.94 ± 2.36	-4.68 ± 1.63
Female	20	0.97 ± 0.80	10.5 ± 8.5	0.58 ± 0.89	5.03 ± 2.29	-4.43 ± 1.84
Total	32	1.10 ± 0.99	12.2 ± 10.1	0.72 ± 1.00	5.28 ± 2.30	-4.51 ± 1.77

Results are expressed as mean ± standard deviation.

**Figure 3:**
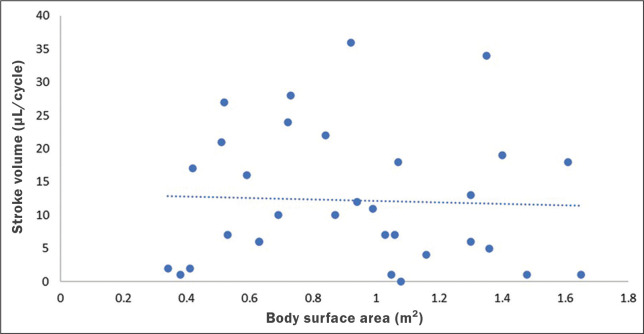
Correlation between BSA and CSF stroke volume (ρ = -0.076; *p* = 0.67).

**Figure 4:**
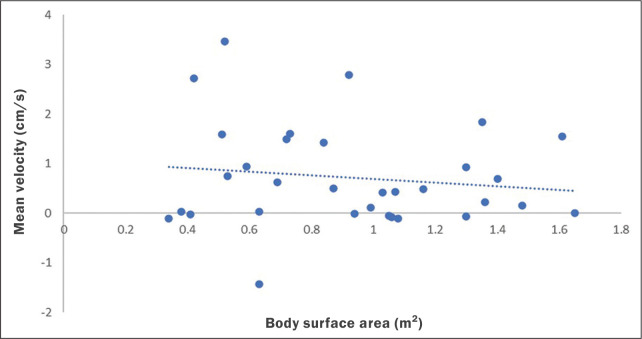
Correlation between BSA and CSF mean velocity (r = -0.079; *p* = 0.67).

No statistically significant differences were found between age groups and sexes regarding any parameter under investigation. Figure 3 describes the correlation between BSA and stroke volume. The Spearman correlation coefficient (ρ) between those variables was -0.076 (*p* = 0.67). Figure 4 shows the correlation between BSA and mean velocity. The Pearson correlation coefficient (r) between those variables was -0.079 (*p* = 0.67).

## DISCUSSION

Traditionally, CSF flow dynamics have been studied through animal experiments and with mathematical models, most of which provide rough and indirect approximations, which may have limited applicability in human subjects^(18)^. The advent of MRI has allowed us to improve our understanding of CSF flow dynamics significantly, in physiological and pathological conditions, in a simple, noninvasive manner. This imaging modality enables us to evaluate multiple flow parameters such as peak velocity, mean velocity, and stroke volume^(2)^. This quantitative evaluation is performed by means of cine-PC MRI sequences, which use a velocity encoding gradient to generate signal contrast between moving and stationary hydrogen atoms^(19)^. Cine-PC MRI is a noninvasive technique that allows the evaluation of flow without requiring the use of ionizing radiation or the injection of contrast medium, making it suitable for use in the pediatric population^(2)^. The main drawback of the technique is that it requires sedation in young children.

There have been few studies evaluating CSF dynamic parameters and their association with age and sex in normal pediatric subjects. Öztürk et al.^(20)^ studied 83 healthy pediatric subjects and found a peak velocity of 5.77 ± 3.18 cm/s and a mean velocity of 0.83 ± 0.73 cm/s. They also found significant differences in peak velocities between adolescents and children, as well as between males and females, although they found no significant differences in mean velocities. Conversely, we found no significant sex- or age-related differences between any of the dynamic CSF parameters evaluated. When making a comparison based on a variable such as age in pediatric studies, it should be borne in mind that the classification may vary depending on the age ranges assigned to each subgroup, which can lead to different reporting of results in comparison with the published literature.

Moreover, literature is also limited in relation to the evaluation of these measurements according to biometric parameters. Demirtaş et al.^(21)^ evaluated CSF flow in 137 children and found no significant correlation between body mass index and CSF flow parameters. Although we hypothesized that mean velocity and stroke volume would correlate with BSA, we found no such correlations. That might be due to minimal variations in the cross-sectional area of the cerebral aqueduct in the pediatric population, which has been reported to be approximately 0.5 mm^2^, compared with 0.8 mm^2^ in adults^(22,23)^. These results indicate that the CSF parameters measured are independent of biometric values.

We consider the present study to be of relevance because it contributes to the limited existing literature regarding MRI evaluation of CSF dynamics in healthy populations. In addition, corresponding studies from Latin America are scarce, and this study may help characterize CSF parameters in the Latin American population. Our study has some limitations. First, there were artifacts in some of the cine-PC MRI sequences, which led to their exclusion from the study and a reduction in the sample size. Second, in some cases, the placement of the region of interest was complicated by the presence of a very narrow cerebral aqueduct, which could increase the margin of error. Finally, neonates were not evaluated. These limitations have also been reported by other authors^(19)^.

## CONCLUSION

We determined the normal values for various parameters of CSF flow dynamics in healthy pediatric subjects, stratifying them by sex and age group, and found no significant differences. We also found that BSA did not correlate with mean velocity or stroke volume.
